# Supplementary material for: Locus-Specific Enrichment Analysis of 5-Hydroxymethylcytosine Reveals Novel Genes Associated with Breast Carcinogenesis
Source: Cells. 2022 Sep 20;11(19):2939. doi: 10.3390/cells11192939 (PMC9562672; doi:10.3390/cells11192939)

SUPPLEMENTARY FIGURES

Figure S1: PCA and HCA of 5-mC and 5-hmC enriched breast tumour and normal tissues

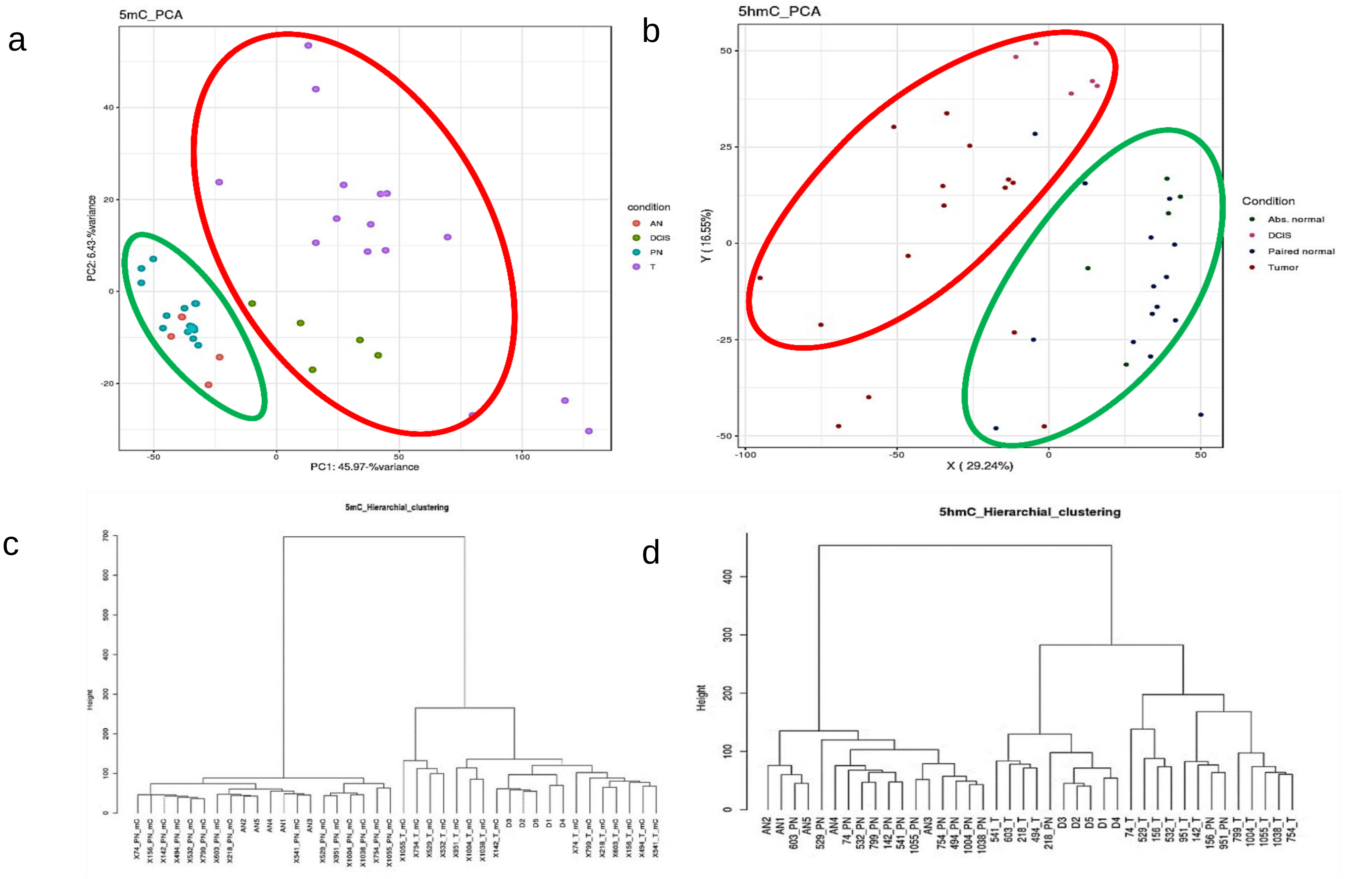

Figure S2: Genome-wide distribution of DhMRs and DMRs

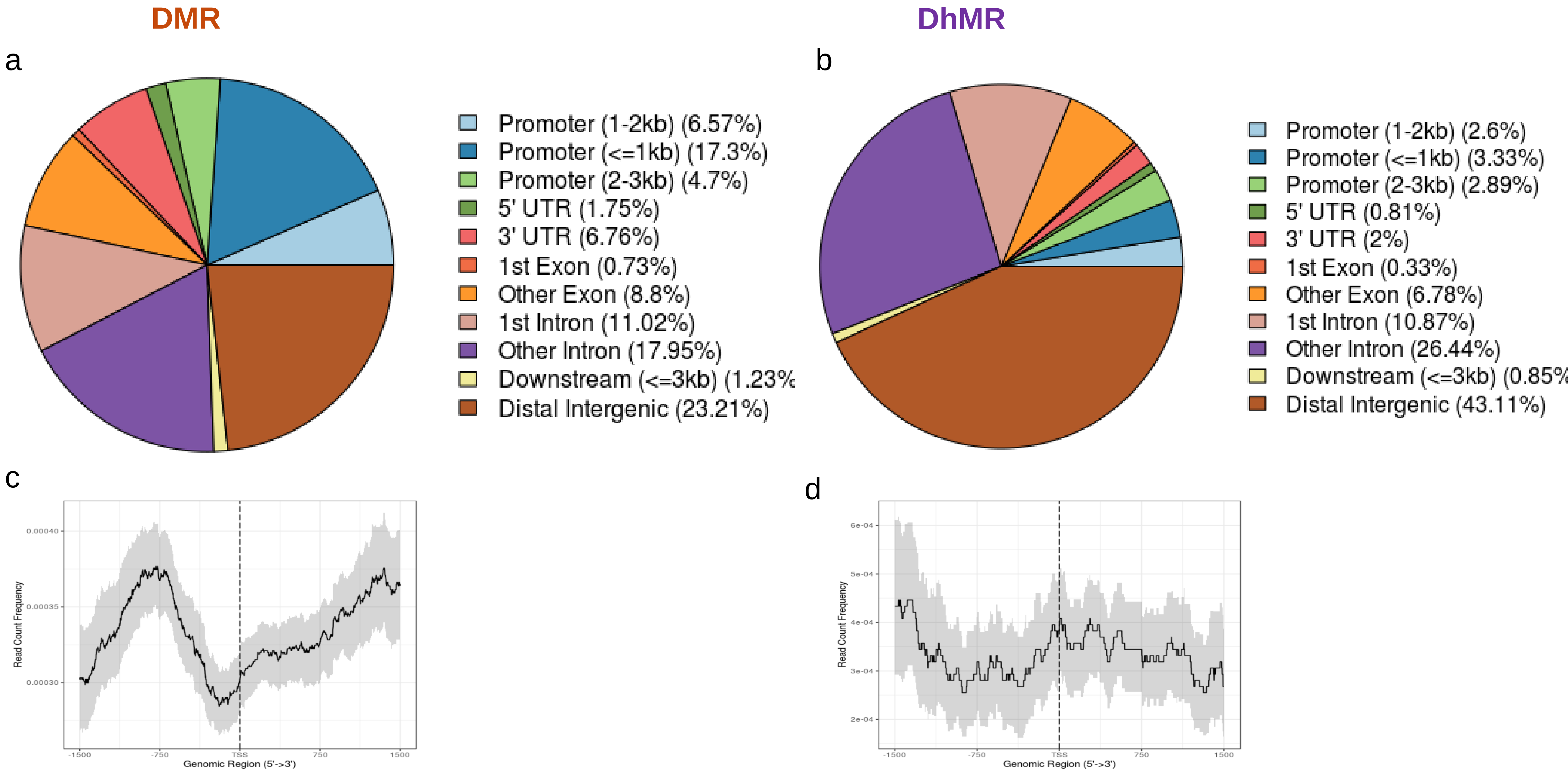

Figure S3. Locus Overlap analysis and ChIP seq analysis using LOLA webtool and UALCAN

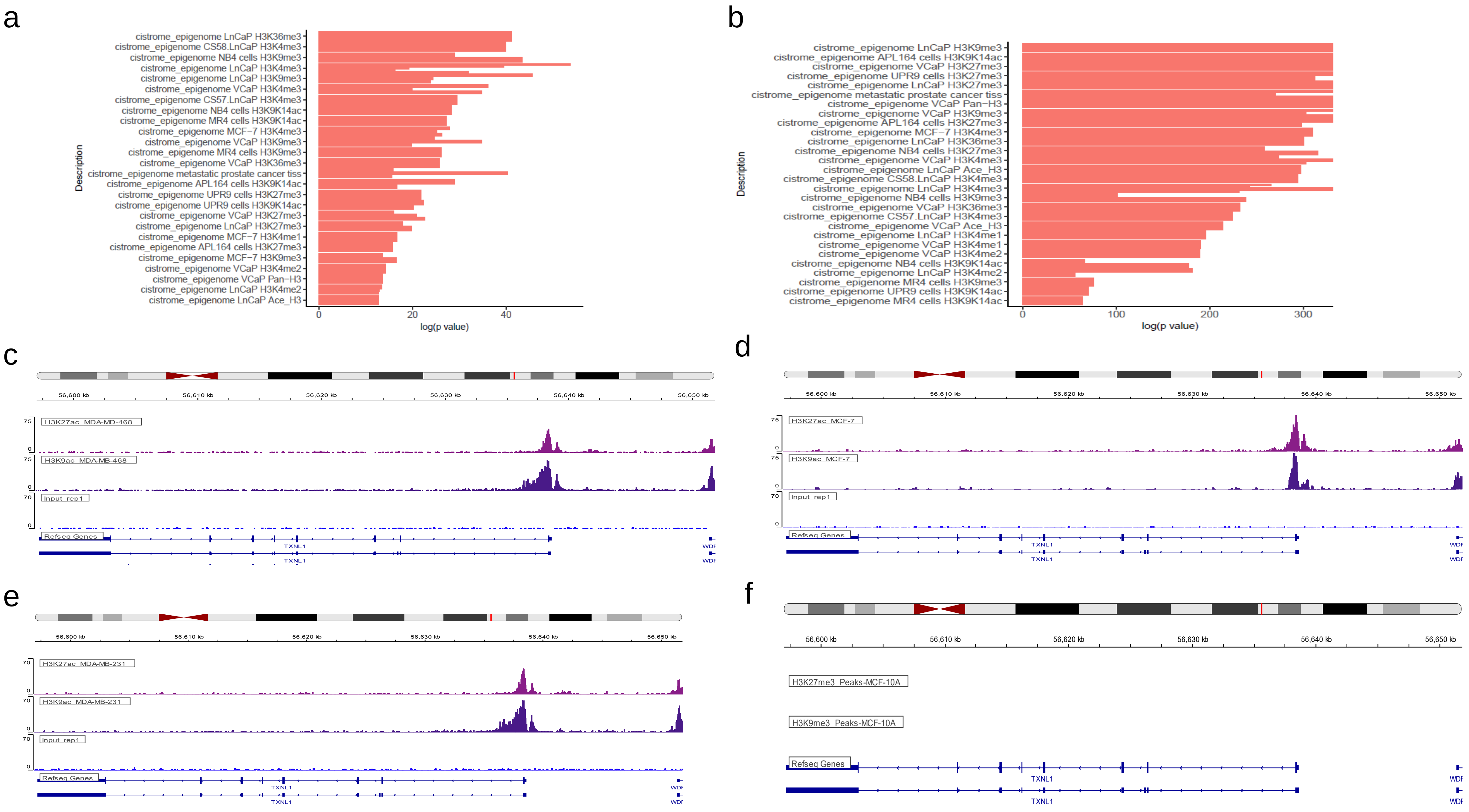

Figure S4: KEGG and GO term pathway enrichment analysis

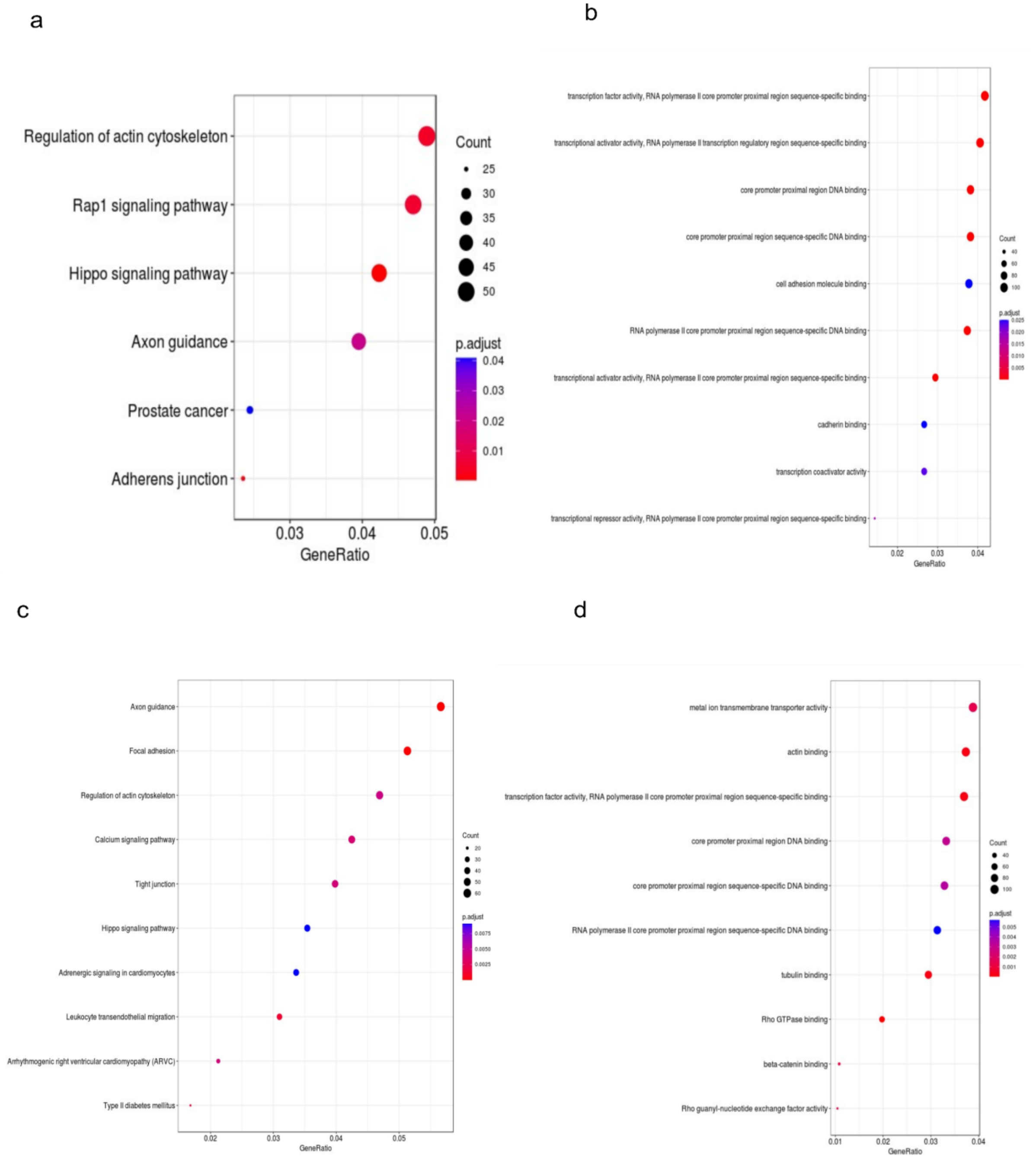

Figure S5. Survival analysis of hyper-hmC candidates

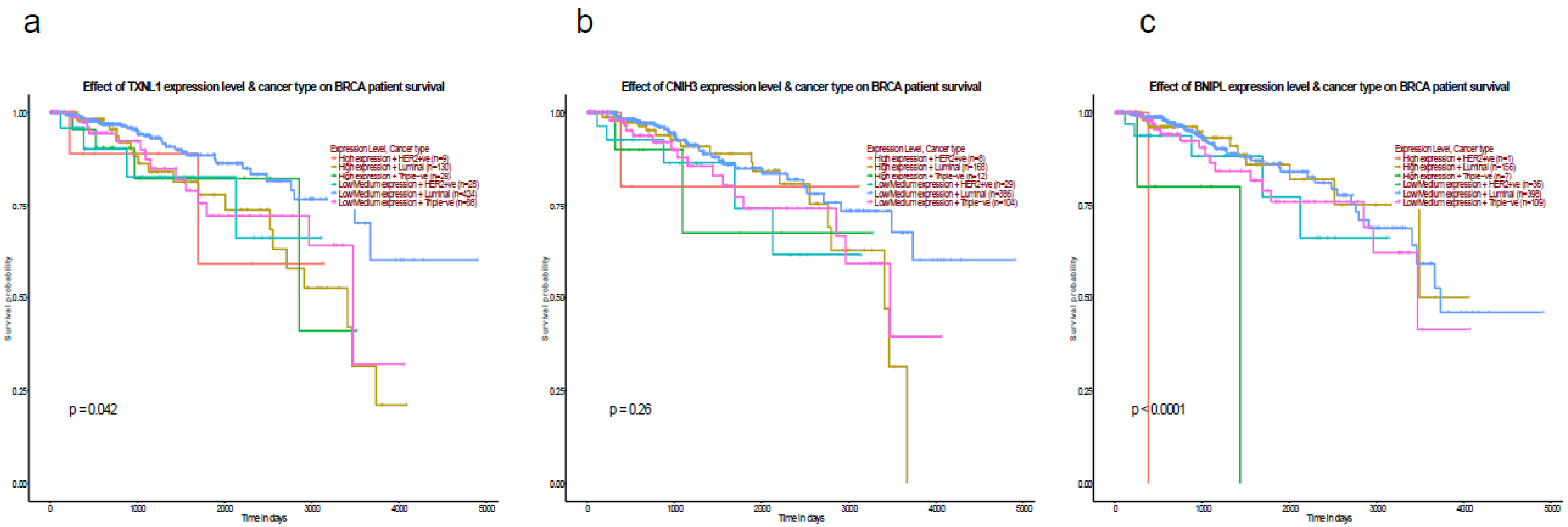

Supplement: Supplementary file 1 [file cells-11-02939-s001.zip › Supplementary figures.pdf]
