# Supplementary material for: Locus-Specific Enrichment Analysis of 5-Hydroxymethylcytosine Reveals Novel Genes Associated with Breast Carcinogenesis
Source: Cells. 2022 Sep 20;11(19):2939. doi: 10.3390/cells11192939 (PMC9562672; doi:10.3390/cells11192939)
Supplement: Supplementary file 1 [file cells-11-02939-s001.zip › Table S1a-b.pdf]

Supplementary Table

Table S1a. Genomic Feature of 5-mC in breast cancer and normal tissues

Genomic Features		Tumour (IDC) (n>=10) (%)	Paired Normal (n>=10) (%)	DCIS (n=5) (%)	Apparent normal (n=5) (%)
**Promoters**	Promoter(>1kb)	8.24	6.09	7.41	6.34
	Promoter(1-2kb)	4.17	3.44	3.76	3.3
	Promoter(2-3kb)	3.68	3.15	3.31	2.88
**UTRs**	5'UTR	1.69	1.32	1.54	1.4
	3'UTR	4.35	3.3	3.89	3.32
**Gene body**	1st exon	0.51	0.4	0.46	0.42
	Other exon	9.41	7.98	8.52	7.72
	1st intron	9.72	9.7	9.44	9.57
	Other intron	24.26	24.64	24.42	24.43
**Distal intergenic**	Downstream (>300kb)	0.16	0.12	0.12	0.12
	Distal intergenic	33.81	39.86	37.13	40.5

Table S1b. Genomic Feature of 5-hmC in breast cancer and normal tissues

Genomic features		Tumour (IDC) (n>=10) (%)	Paired Normal (n>=10) (%)	DCIS (n=5) (%)	Apparent normal (n=5) (%)
**Promoters**	Promoter(>1kb)	24.89	30.89	48.74	6.25
	Promoter(1-2kb)	2.46	2.3	1.88	
	Promoter(2-3kb)	2.6	2.69	2.06	6.25
**UTRs**	5'UTR	1.46	1.57	0.97	
	3'UTR	3.64	3.99	2.64	
**Gene body**	1st exon	0.9	0.88	1.12	12.5
	Other exon	5.8	6.52	3.44	25
	1st intron	7.73	6.63	4.61	6.25
	Other intron	14.37	12.66	7.02	18.75
**Distal intergenic**	Downstream (>300kb)	0.08	0.08	0.13	
	Distal intergenic	35.98	31.8	27.4	25
